# Cigarette smoke-induced pulmonary emphysema in *scid*-mice. Is the acquired immune system required?

**DOI:** 10.1186/1465-9921-6-147

**Published:** 2005-12-16

**Authors:** An I D'hulst, Tania Maes, Ken R Bracke, Ingel K Demedts, Kurt G Tournoy, Guy F Joos, Guy G Brusselle

**Affiliations:** 1 Department of Respiratory Diseases, Ghent University Hospital, Ghent, Belgium

## Abstract

**Background:**

Chronic obstructive pulmonary disease is associated with a chronic inflammatory response of the host to chronic exposure to inhaled toxic gases and particles. Although inflammatory cells of both the innate and adaptive immune system infiltrate the lungs in pulmonary emphysema and form lymphoid follicles around the small airways, the exact role of the acquired immune system in the pathogenesis of emphysema is not known.

**Methods:**

In this study, wild type *Balb/c *mice and immunodeficient *scid *mice – which lack functional B- and T-cells – were exposed to mainstream cigarette smoke (CS) for 5 weeks or 6 months.

**Results:**

Subacute CS-exposure for 5 weeks significantly increased innate inflammatory cells (neutrophils, macrophages and dendritic cells) in the bronchoalveolar lavage (BAL) fluid of wild type mice and *scid *mice, which correlated with the CS-induced upregulation of the chemokines Monocyte Chemotactic Protein-1, Macrophage Inflammatory Protein-3α and KC (= mouse Interleukin-8). Chronic CS-exposure for 6 months significantly increased the number of neutrophils, macrophages, dendritic cells, CD4^+ ^and CD8^+ ^T-lymphocytes in BAL fluid and lungs of wild type mice compared to air-exposed littermates, and augmented the size and number of peribronchial lymphoid follicles. In contrast, neither B-lymphocytes, nor T-lymphocytes, nor lymphoid follicles could be discerned in the lungs of air- or CS-exposed *scid *mice. Importantly, chronic CS-exposure induced pulmonary emphysema in both wild type animals and *scid *mice, as evidenced by a significant increase in the mean linear intercept and the destructive index of CS-exposed versus air-exposed animals. The CS-induced emphysema was associated with increased mRNA expression of matrix metalloproteinase-12 in the lungs and increased protein levels of Tumor Necrosis Factor-α in the BAL fluid of CS-exposed *Balb/c *and *scid *mice compared to air-exposed littermates.

**Conclusion:**

This study suggests that the adaptive immune system is not required per se to develop pulmonary emphysema in response to chronic CS-exposure, since emphysema can be induced in *scid *mice, which lack lymphoid follicles as well as functional B- and T-cells.

## Background

Chronic obstructive pulmonary disease (COPD) is currently listed as the fifth leading cause of death in the world, and is also an important cause of chronic disability and permanent impairment, representing a major economic and social burden worldwide [1,2]. COPD is defined by the Global Initiative for chronic Obstructive Lung Disease (GOLD) as "a disease state characterized by airflow limitation that is not fully reversible, and that is usually both progressive and associated with an abnormal inflammatory response of the lungs to noxious particles or gases" [3]. Cigarette smoking is by far the most important risk factor for COPD. However, only a susceptible minority (± 15–20%) of tobacco smokers develops clinically significant COPD, suggesting that genetic factors (such as the rare hereditary deficiency of α1-antitrypsin) must modify each individual's risk. Therefore, although the major environmental risk factor for COPD – tobacco smoke – is well known since many years, the cellular and molecular mechanisms that are involved in the pathogenesis of COPD have not yet been fully elucidated.

In COPD, there is a chronic inflammation of the small airways and the lung parenchyma, leading to fixed narrowing of small airways and alveolar wall destruction (emphysema) [4]. Multiple studies of lung specimens, bronchial biopsies and bronchoalveolar lavage fluid of patients with COPD have demonstrated that this chronic inflammation is characterized by increased numbers of alveolar macrophages, neutrophils and lymphocytes [5]. Especially CD8^+ ^T-lymphocytes are increased in the peripheral airways and lungs of smokers with COPD as compared with asymptomatic smokers with normal lung function [6,7]. Moreover, the extent of emphysema in smokers has been related to the number of CD3^+ ^T-cells in the alveolar wall, and CD8^+ ^T-lymphocytes appeared to be the predominant cells in the alveolar wall of emphysematous lungs [8,9]. One of the important functions of CD8^+ ^T-cells is their cytolytic activity, inducing cell death by perforin mediated lysis and apoptosis by caspase activation [10]. These data suggest an important role of CD8^+ ^T-lymphocytes in the pathogenesis of COPD and emphysema.

Recently, Hogg et al. further characterized the nature of the small airway obstruction in patients with COPD of increasing severity [11]. Progression of COPD from mild disease (GOLD stage 1) to very severe COPD (GOLD stage 4) was associated with thickening of the airway wall and with an increased number of airways containing lymphocytes (not only CD8^+ ^T-cells, but also CD4^+ ^T-cells and B-cells). Interestingly, these T- and B-lymphocytes are not only increased in numbers, but are also organized into follicles, especially in patients with severe (GOLD stage 3) and very severe (GOLD stage 4) COPD [11]. These data suggest that an adaptive immune response develops in COPD patients, but it is not known whether this response is elicited by the toxic particles and gases present in cigarette smoke, or develops in relation to the microbial colonization and infection known to occur in the later stages of COPD. Moreover, it is not known whether the adaptive immune response (including CD8^+ ^T-cells and B-cells) is required for the development of pulmonary emphysema. Recently, we characterized the time course of cigarette smoke-induced pulmonary inflammation in a murine model of COPD [12]. In the present paper, we report the effects of subacute (5 weeks) or chronic (24 weeks) exposure to cigarette smoke in severe combined immunodeficiency (*scid*) mice, which lack functional T-and B-cells, but exhibit normal differentiation and function of myeloid cells, antigen presenting cells and natural killer cells [13,14]. We analysed the inflammatory responses in both the bronchoalveolar lavage (BAL) and pulmonary compartment, and we performed morphometric analysis to quantify the extent of emphysema in *scid *mice and wild type animals, exposed to cigarette smoke or control air for 6 months.

## Methods

### Animals

Homozygous male Fox Chase C.B17-*scid *mice (8 weeks of age) were obtained from M&B ((Møllegaard and Bomholtgård Breeding and Research Center A/S, Ry, Denmark). Wild type *Balb/c *mice were used as control mice, as suggested by M&B. All mice were housed in sterilized cages with filter tops and received sterilized food and water ad libitum. The local Ethics Committee for animal experimentation of Ghent University (Ghent, Belgium) approved all *in vivo *manipulations.

### Smoke exposure

Mice (n = 8 per group) were exposed to cigarette smoke (CS), as described previously [12]. Briefly, groups of 8 mice were exposed to the tobacco smoke of 5 cigarettes (Reference Cigarette 2R4F without filter, University of Kentucky, Lexington, KY) four times a day with 30 minutes smoke-free intervals, 5 days a week for 5 and 24 weeks (1 and 6 months). During the first week of the experiment, mice were exposed to the smoke of only one cigarette a day to acclimatize them to the higher dose during the second week. The smoke:air ratio used in this study was 1:6. The control group was exposed to air. Carboxyhemoglobin levels in serum of smoke-exposed mice was 8.3 ± 1.4 % vs 1.0 ± 0.2 % in air-exposed mice (n = 7).

### Bronchoalveolar lavage (BAL)

24 hours after the last smoke exposure mice were weighed and sacrificed with an overdose of pentobarbital (Sanofi, Libourne, France) and a tracheal cannula was inserted. 1 ml of Hank's balanced salt solution (HBSS), free of ionized calcium and magnesium but supplemented with 0.05 mM sodium EDTA, was instilled 4 times via the tracheal cannula and recovered by gentle manual aspiration. The 4 lavage fractions were centrifuged, the cell pellet was washed twice and finally resuspended in 1 ml of HBSS. A total cell count was performed in a Bürcker chamber and the differential cell counts (on at least 400 cells) were performed on cytocentrifuged preparations using standard morphologic criteria after May-Grünwald-Giemsa staining. Flow cytometric analysis of BAL-cells was also performed.

### Preparation of lung single cell suspensions

Following BAL, the pulmonary and systemic circulation was rinsed. The left lung was used for histology, the right lung for the preparation of a cell suspension as detailed previously [15]. Briefly, the lung was thoroughly minced, digested, subjected to red blood cell lysis, passed through a 50 μm cell strainer, and kept on ice until labelling. Cell counting was performed with a Z2 Beckman-Coulter particle counter (Beckman-Coulter, Ghent, Belgium).

### Labelling of BAL-cells and lung single cell suspensions for flow cytometry

Cells were pre-incubated with F_c_-receptor blocking antibody (anti-CD16/CD32, clone 2.4G2) to reduce non-specific binding. Monoclonal antibodies used to identify mouse DC populations were: biotinylated anti-CD11c (N418) and phycoerythrin (PE)-conjugated anti-I-E^k ^(14-4-4S). The following antibodies were used to stain mouse T-cell subpopulations: fluorescein isothiocyanate (FITC)-conjugated anti-CD4 (L3T4), FITC-conjugated anti-CD8 (Ly-2), and biotinylated anti-CD3 (145-2C11) monoclonal antibodies. The additional marker used for activation was PE-conjugated anti-CD69 (H1.2F3). Antibodies used to characterize B-lymphocytes were: PE-conjugated anti-CD19 (1D3) together with anti-CD11c. Biotinylated anti-CD11c and anti-CD3 were revealed by incubation with streptavidine-allophycocyanine (SAv-APC). All monoclonal antibodies were obtained from BD Pharmingen (Erembodegem, Belgium), except anti-CD11c (N418 hybridoma, a gift from Prof. M. Moser, Brussels Free University, Belgium).

As a last step before analysis, cells were incubated with 7-amino-actinomycin (7-AAD or viaprobe, BD Pharmingen) for dead cell exclusion. All labelling reactions were performed on ice in FACS-EDTA buffer.

Flow cytometry data acquisition was performed on a dual-laser FACS Vantage™ flow cytometer running CELLQuest™ software (Becton Dickinson, Mountain View, California). FlowJo software  was used for data analysis.

### Histology

The left lung was fixated by gentle infusion of fixative (4% paraformaldehyde) through the tracheal cannula [12]. After excision, the lung was immersed in fresh fixative during 2 h. The lung lobe was embedded in paraffin and cut in 3 μm transversal sections. Lung tissue samples were stained with hematoxylin and eosin, and examined by light microscopy for histological sections. For each animal, 10 fields at a magnification of 200× were captured randomly from the 4 different zones of the left lung (upper, middle upper, middle basal and basal zone) by lab technicians using a Zeiss KS400 image analyzer platform (KS400, Zeiss, Oberkochen, Germany).

### Quantification of emphysema

Emphysema is a structural disorder characterized by destruction of the alveolar walls and enlargement of the alveolar spaces. We determined enlargement of alveolar spaces by quantifying the mean linear intercept (L_m_) and destruction of alveolar walls by measuring the destructive index (DI) in air- and CS-exposed mice (n = 7 for air-exposed *Balb/c *and *scid *mice and CS-exposed *Balb/c *mice; n = 6 for CS-exposed *scid *mice), as described previously [12,16,17].

Quantification of airspace enlargement was determined after 6 months smoke exposure by measuring the mean linear intercept (L_m_), using image analysis software (Image J 1.33). Only sections without cutting artefacts, compression or hilar structures (airway or blood vessel with a diameter larger than 50 μm) were used in the analysis. The L_m _was measured by placing a 100 × 100 μm grid over each field. The total length of each line of the grid divided by the number of alveolar intercepts gives the average distance between alveolated surfaces, or the L_m _[16].

The destruction of alveolar walls was quantified by the destructive index (DI) [17]. A grid with 42 points that were at the center of hairline crosses was superimposed on the lung field. Structures lying under these points were classified as normal (N) or destroyed (D) alveolar and/or duct spaces. Points falling over other structures, such as duct walls, alveolar walls, etc. did not enter into the calculations. The DI was calculated from the formula: DI = D / (D + N) × 100.

Two investigators – blinded to the exposure status and the genotype of the mice – independently measured DI and L_m _(interobserver correlation of r = 0.85, p < 0.001).

### Morphometric quantification of lymphoid follicles

To evaluate the presence of lymphoid follicles in lung tissue after 6 months smoke exposure (n = 7 for air-exposed *Balb/c *and *scid *mice and CS-exposed *Balb/c *mice; n = 6 for CS-exposed *scid *mice), lung sections obtained from formalin-fixed, paraffin-embedded lung lobes were subjected to the following immunohistological stainings: (A) B220 staining and (B) CD3/B220 doublestaining. (A) Sections were stained with anti-B220-biotin (BD Pharmingen) after Boehringer blocking (with triton) (DakoCytomation, Heverlee, Belgium). Then slides were incubated with streptavidin horseradish peroxidase and colored with diaminobenzidine (DAB) (both obtained from DakoCytomation). (B) At first, sections were incubated with Boehringer blocking reagent with triton and primary antibody anti-CD3, followed by goat-anti-rabbit biotin (both obtained from DakoCytomation). Then, slides were incubated with streptavidin horseradish peroxidase and colored with DAB. In a second step, sections were stained with anti-B220-biotin after Boehringer blocking (with triton). Finally, slides were incubated with streptavidin alkaline phosphatase (DakoCytomation) and colored with Vector blue (Vector Laboratories, Inc., Burlingame, CA). Lymphoid follicles were counted in the tissue area surrounding the airways (airway perimeter 0–2000 μm). Results were expressed as counts relative to the numbers of airways per lung section.

### Measurement of immunoglobulins (Ig)

After 1 and 6 months CS-exposure, IgG and IgM concentrations in serum were determined using a commercially available ELISA kit (R&D Systems Europe, Abingdon, UK).

### Measurement of cytokines and chemokines

After 6 months CS-exposure, Macrophage Inflammatory Protein-3α (MIP-3α) and KC (mouse IL-8) protein levels were determined in BAL-fluid using commercially available ELISA kits (R&D Systems Europe, Abingdon, UK).

After 6 months CS-exposure, Interleukin-6 (IL-6), Interleukin-10 (IL-10), Monocyte Chemotactic Protein-1 (MCP-1), Interferon-γ (IFN-γ), Tumor Necrosis Factor-α (TNF-α) and Interleukin-12 (IL-12p70) in BAL-fluid were determined by FACS using the Cytometric Bead Array (CBA – Mouse inflammation kit; BD Biosciences, San Diego, CA, USA), following the manufacturers instructions. A mixture of 6 capture bead populations, each with distinct fluorescence intensities and coated with antibodies specific for IL-6, IL-10, MCP-1, IFN-γ, TNF-α and IL-12p70, were prepared. The CBA capture beads were incubated together with PE-conjugated detection antibodies, test samples or standards, to form sandwich complexes. Following acquisition of sample data using the flow cytometer (FACSCalibur™ flow cytometer, BD Biosciences), the sample results were generated in graphical and tabular format using the CBA Analysis Software (BD Biosciences, San Diego, CA, USA).

### Semiquantitative RT-PCR analysis

Total lung RNA was extracted using the RNeasy Midi kit (Qiagen, Hilden, Germany), with an additional DNAse-step. Reverse transcription was performed at 48°C for 30 min followed by 12 min incubation at 95°C for denaturation of RNA-DNA heteroduplexes, and a DNA-amplification with 50 cycles of 95°C for 15 sec and 60°C for 1 min. RT-PCR was performed starting from 10 ng of total RNA, using an ABI PRISM 7700 Sequence Detection System (Applied Biosystems, USA). After 1 month CS-exposure, expression of matrix metalloproteinase-12 (MMP-12) mRNA, perforin mRNA, granzyme B mRNA and Macrophage Inflammatory Protein-3α (MIP-3α) mRNA relative to hypoxanthine guanine phophoribosyl transferase (hprt) mRNA, was analysed with the Assays-on-Demand™ Gene Expression Products (Applied Biosystems, USA).

### Statistical analysis

Reported values are expressed as mean ± standard error of the mean (SEM). Statistical analysis was performed with Sigma Stat software (SPSS 11.0 Inc, Chicago, IL, USA) using non-parametric tests (Kruskall-Wallis, Mann-Whitney U). P-values under 0.05 were considered as significant.

## Results

### Cigarette smoke exposure increases innate inflammatory cells in the bronchoalveolar lavage fluid of both wild type mice and *scid *mice

**Figure 1:**
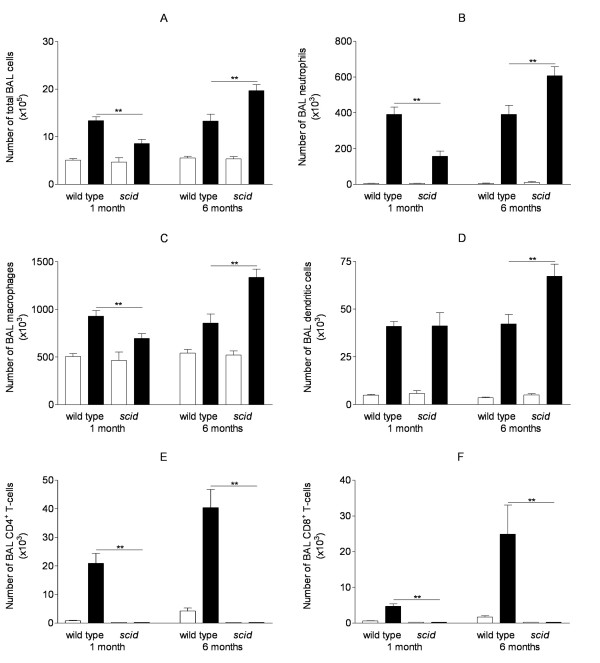
**Cell differentiation in bronchoalveolar lavage fluid upon air or cigarette smoke exposure**. Effect of cigarette smoke (CS) exposure for 1 month or 6 months on cell differentiation in bronchoalveolar lavage fluid (absolute numbers in BAL fluid) in wild type mice and *scid *mice (air-exposed mice: open bar, CS-exposed mice: closed bar): (A) total BAL cells, (B) neutrophils, (C) macrophages, (D) dendritic cells, (E) CD4^+ ^T-cells and (F) CD8^+ ^T-cells. Animals (n = 8) were exposed to 5 cigarettes/group/exposure; 4 exposures/day; 5 days/week. Results are expressed as means ± SEM (** p < 0.001 versus wild type mice at corresponding timepoint). Numbers of total BAL cells, neutrophils, macrophages, dendritic cells, CD4^+ ^and CD8^+ ^T-cells are significantly increased in CS-exposed versus air-exposed wild type mice and *scid *mice. No CD4^+ ^and CD8^+ ^T-cells were detected in BAL of *scid *mice.

Subacute exposure to cigarette smoke (CS) for 1 month significantly increased the absolute number of total cells, neutrophils, macrophages and dendritic cells in the BAL fluid of wild type animals compared to air-exposed littermates (see figure 1A–D). Also after chronic CS-exposure for 6 months, there is a significant increase in the number of neutrophils, macrophages and dendritic cells in the BAL compartment of wild type mice in comparison with air-exposed littermates. In *scid *mice, both subacute and chronic exposure to CS significantly increased the number of BAL fluid neutrophils, macrophages and dendritic cells compared to air exposure for 1 and 6 months, respectively (figure 1A–D). Subacute CS-exposure induced a larger increase in the absolute number of BAL cells, neutrophils and macrophages in wild type animals compared to *scid *mice (p < 0.05). On the other hand, chronic exposure to CS induced an even larger increase in the numbers of neutrophils, macrophages and dendritic cells in *scid *mice compared to wild type animals (p < 0.001).

Also in the lung parenchyma, chronic exposure to CS increased significantly the number of macrophages and dendritic cells in wild type mice and *scid *mice (number of pulmonary macrophages: wild type: air = 76.75 ± 13.2 × 10^4 ^versus CS = 152.73 ± 6.23 × 10^4^, p = 0.002; *scid*: air = 53.33 ± 4.20 × 10^4 ^versus CS = 81.35 ± 3.02 × 10^4^, p = 0.015; number of pulmonary dendritic cells: wild type: air = 53.54 ± 1.72 × 10^4 ^versus CS = 83.14 ± 2.70 × 10^4^, p = 0.006; *scid*: air = 40.80 ± 3.12 × 10^4 ^versus CS = 103.55 ± 2.10 × 10^4^, p = 0.003).

### Chronic cigarette smoke exposure increases the number of B- and T-lymphocytes in the lungs of wild type animals; in contrast, lymphocytes remain absent in the lungs of *scid *mice

In wild type animals, subacute CS-exposure for 1 month induced a significant increase in the number of both CD4^+ ^and CD8^+ ^lymphocytes in BAL fluid compared to air-exposed littermates (figure 1E and 1F). Chronic exposure to CS for 6 months further increased the number of BAL CD4^+ ^and CD8^+ ^T-lymphocytes in wild type mice. In contrast, neither CD4^+ ^nor CD8^+ ^T-lymphocytes could be detected in the BAL fluid of *scid *mice after air or CS-exposure for 1 and 6 months (figure 1E and 1F).

**Figure 2:**
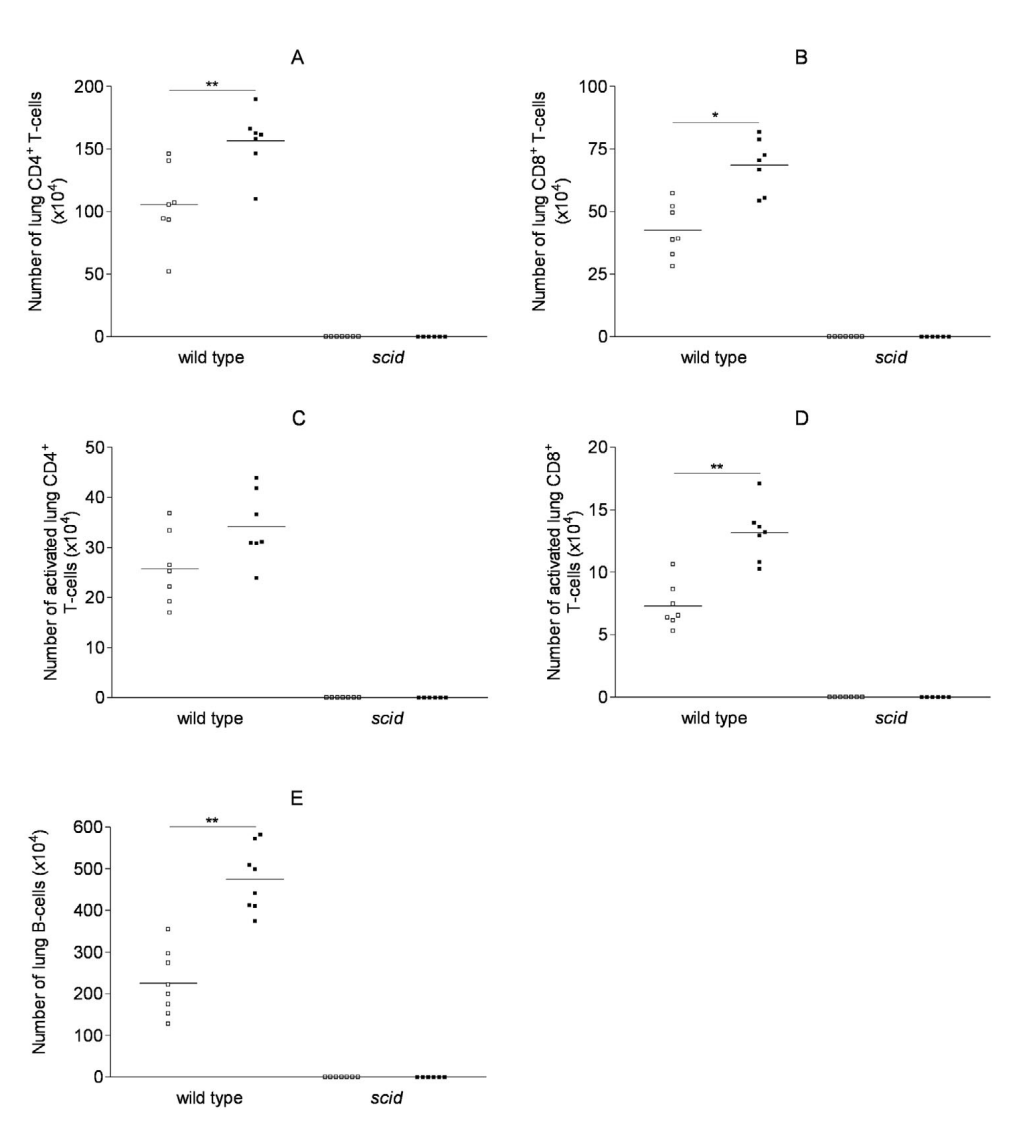
**T- and B-cell populations in the lungs upon air or cigarette smoke exposure**. Effect of cigarette smoke (CS) exposure for 6 months on T-cell populations in the lungs (absolute numbers in the lungs; determined by flow cytometry) in wild type mice and *scid *mice (air-exposed mice: open squares, CS-exposed mice: closed squares): (A) lung CD4^+ ^T-lymphocytes, (B) lung CD8^+ ^T-lymphocytes, (C) activated lung CD4^+ ^T-lymphocytes, (D) activated lung CD8^+ ^T-lymphocytes and (E) lung B-lymphocytes. Animals (n = 8) were exposed to 5 cigarettes/group/exposure; 4 exposures/day; 5 days/week (* p < 0.05 and ** p < 0.001 versus air-exposed mice).

**Figure 5:**
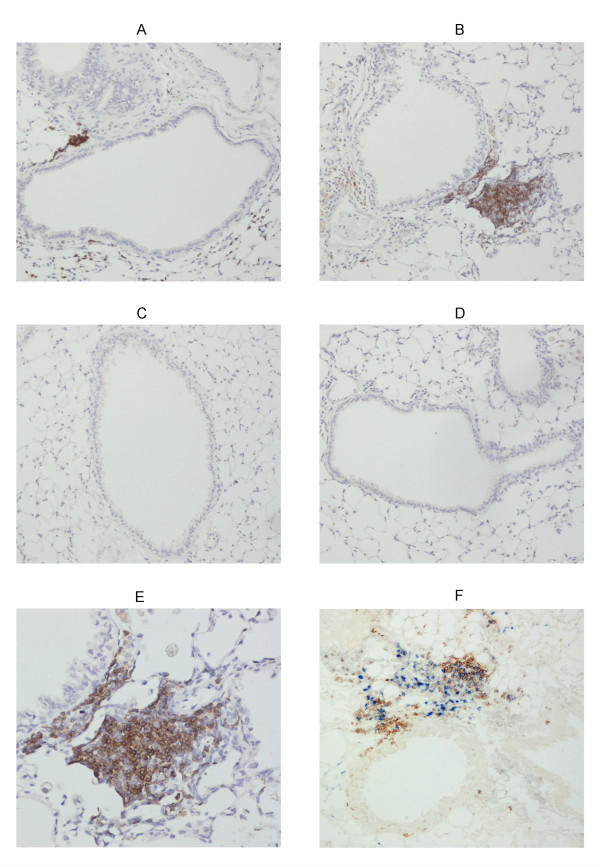
**Lymphoid follicles in the lungs upon air or cigarette smoke exposure**. Photomicrographs of lymphoid follicles in lungs of air- and cigarette smoke (CS)-exposed wild type mice and *scid *mice at 6 months (magnification × 100). (A)-(E) B220 staining (brown = B220 positive cells): (A) air-exposed wild type mice, (B) CS-exposed wild type mice, (C) air-exposed *scid *mice, (D) CS-exposed *scid *mice and (E) CS-exposed wild type mice (magnification × 200). (F) CD3/B220 staining (brown = CD3 positive cells; blue = B220 positive cells): CS-exposed wild type mice.

In lung digests, chronic CS-exposure significantly increased the number of B-lymphocytes, CD4^+ ^T-lymphocytes, CD8^+ ^T-lymphocytes and activated CD8^+ ^T-cells in wild type mice compared to air-exposed littermates (figure 2A–E). In air-exposed as well as CS-exposed *scid *mice, no B-lymphocytes, CD4^+ ^T-cells or CD8^+ ^T-cells could be detected in the lung digests by FACS analysis (figure 2A–E) or in the lung parenchyma by immunohistochemistry (figure 5).

### Chronic cigarette smoke exposure induces pulmonary emphysema in wild type mice and *scid *mice

**Figure 3:**
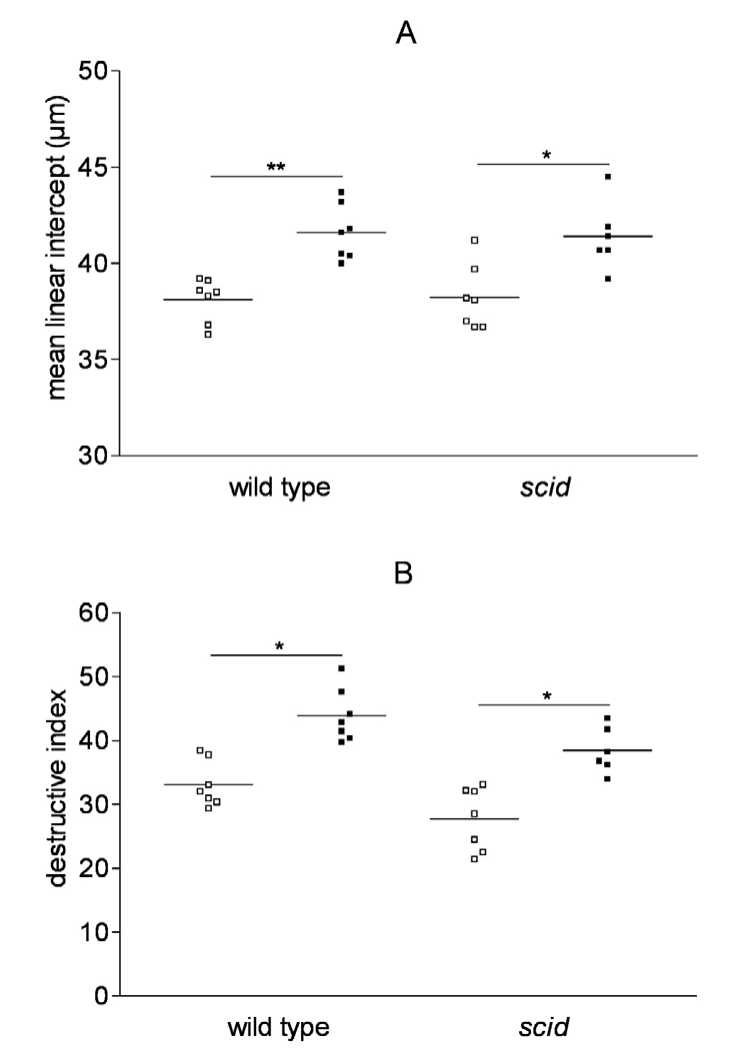
**Quantification of pulmonary emphysema in wild type mice and *scid *mice**. Effect of cigarette smoke (CS) exposure for 6 months on emphysema in wild type mice and *scid *mice (air-exposed mice: open squares, CS-exposed mice: closed squares). (A) mean linear intercept (μm) and (B) destructive index (* p < 0.05 and ** p < 0.01 versus air-exposed mice).

**Figure 4:**
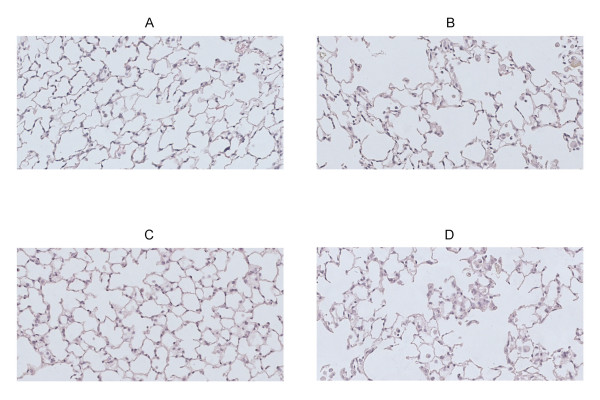
**Pulmonary emphysema in wild type versus *scid *mice after 6 months air or CS-exposure**. Photomicrographs of hematoxylin and eosin stained lung tissue of air- and cigarette smoke (CS)-exposed wild type mice and *scid *mice at 6 months (magnification × 200). (A) air-exposed wild type mice, (B) CS-exposed wild type mice, (C) air-exposed *scid *mice and (D) CS-exposed *scid *mice.

Pulmonary emphysema is characterized by destruction of the alveolar walls due to damage to the lung parenchyma, leading to enlargement of the alveolar spaces. Therefore, to quantify emphysematous lesions it is recommended to evaluate both the airspace enlargement (quantified by measurement of the mean linear intercept [L_m_]) and the destruction of the alveolar walls (quantified by measurement of the destructive index [DI]). Chronic exposure to CS clearly induced pulmonary emphysema in wild type animals, as evidenced by a significant increase in the L_m _(air = 38.1 ± 0.1 μm versus CS = 41.6 ± 0.2 μm, p < 0.01; figure 3A) and in the DI (air = 33.0 ± 0.4 versus CS = 43.8 ± 0.5, p < 0.05; figure 3B) compared to air-exposed wild type littermates. In *scid *mice, CS-exposure for 6 months also significantly increased the L_m _(air = 38.3 ± 0.2 μm versus CS = 41.4 ± 0.2 μm, p < 0.05) and the destructive index (air = 27.7 ± 0.6 versus CS = 37.8 ± 0.8, p < 0.05) compared to air exposure (figure 3A and 3B). The significant airspace enlargement due to chronic CS-exposure in both wild type and *scid *mice is clearly demonstrated on hematoxylin and eosin-stained lung tissue sections in figure 4.

### Serum immunoglobulin levels in wild type mice and *scid *mice

Since up to 15% of *scid *mice may express detectable serum immunoglobulins (Ig) as they become "leaky" upon aging [18], we determined levels of IgG and IgM in serum of *Balb/c *and *scid *mice. At 6 months, air- and CS-exposed wild type animals had similar serum IgG and IgM levels (IgG: air = 600.7 ± 65.9 μg/ml versus CS = 646.3 ± 56.5 μg/ml, N.S.; IgM: air = 146.8 ± 9.6 μg/ml versus CS = 148.0 ± 3.4 μg/ml, N.S.). At 6 months, serum IgG and IgM levels remained below the detection limit in all *scid *mice, whether exposed to air or to CS. Only one *scid *mouse in the CS-exposed group appeared to be leaky, since the serum IgG exceeded 20 μg/ml.

### Chronic cigarette smoke exposure induces pulmonary lymphoid follicles in wild type mice, in contrast to *scid *mice

Immunohistochemistry using anti-CD3 and anti-B220 monoclonal antibodies to stain T-lymphocytes and B-lymphocytes respectively, revealed the presence of only a few small lymphoid follicles in the lung areas surrounding the airways of air-exposed wild type mice (figure 5). Chronic CS-exposure significantly increased the number of peribronchial lymphoid follicles in the lungs of wild type animals (number of lymphoid follicles/airway: air: 0.10 ± 0.01 versus CS: 0.39 ± 0.02, p < 0.001). Moreover, the density of these peribronchial lymphoid follicles was also clearly augmented in wild type animals by CS-exposure (figure 5B,E and 5F). In contrast, no lymphoid follicles could be discerned in the lung parenchyma of *scid *mice, which were exposed to either air or CS for 6 months (figure 5C and 5D).

### Cigarette smoke exposure increases MMP-12 mRNA in the lungs of wild type mice and *scid *mice

**Figure 6:**
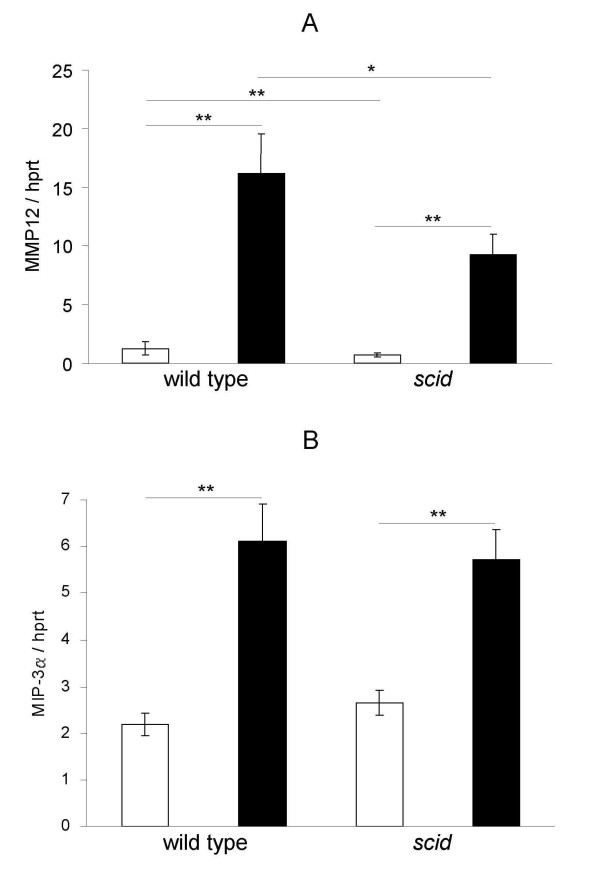
**Expression of MMP-12 and MIP-3α RNA in lungs upon air or cigarette smoke exposure**. (A) MMP-12 and (B) MIP-3α RNA expression in lung tissue of wild type mice and *scid *mice to air or cigarette smoke (CS) exposure for 1 month (air-exposed mice: open bar, CS-exposed mice: closed bar). RT-PCR results are expressed as a ratio target mRNA with hprt (hypoxanthine guanine phosphoribosyl transferase) mRNA (mean ± SEM, n = 5 animals per group, * p < 0.05 and ** p < 0.01). MMP-12: matrix metalloproteinase-12; MIP-3α: Macrophage Inflammatory Protein-3α.

To elucidate the possible mechanisms by which pulmonary emphysema develops in CS-exposed *(scid) *mice, we measured mRNA levels of matrix metalloproteinase-12 (MMP-12), perforin and granzyme B in the lungs of air- and CS-exposed animals by RT-PCR. CS-exposure for one month increased the lung mRNA of MMP-12 in both wild type and *scid *mice compared to air-exposed animals (p < 0.01; figure 6A). In contrast, exposure to CS did not influence the pulmonary mRNA levels of perforin and granzyme B in wild type and *scid *mice (N.S.; data not shown). Interestingly, the baseline mRNA levels for granzyme B in the lungs of air-exposed *scid *mice appeared to be higher than in air-exposed wild type mice (p < 0.01).

Since subacute CS-exposure induces increased numbers of dendritic cells in BAL fluid and lungs of both wild type and *scid *mice, we investigated the mRNA expression of MIP-3α, which is an epithelial-derived chemokine with specific chemoattracting properties towards immature dendritic cells. Exposure to CS for 1 month indeed upregulated the MIP-3α mRNA expression in wild type animals as well as *scid *mice (p < 0.01; figure 6B).

### Cigarette smoke exposure increases protein levels of TNF-α, MCP-1, MIP-3α and KC in bronchoalveolar lavage (BAL) fluid in wild type mice and *scid *mice

**Figure 7:**
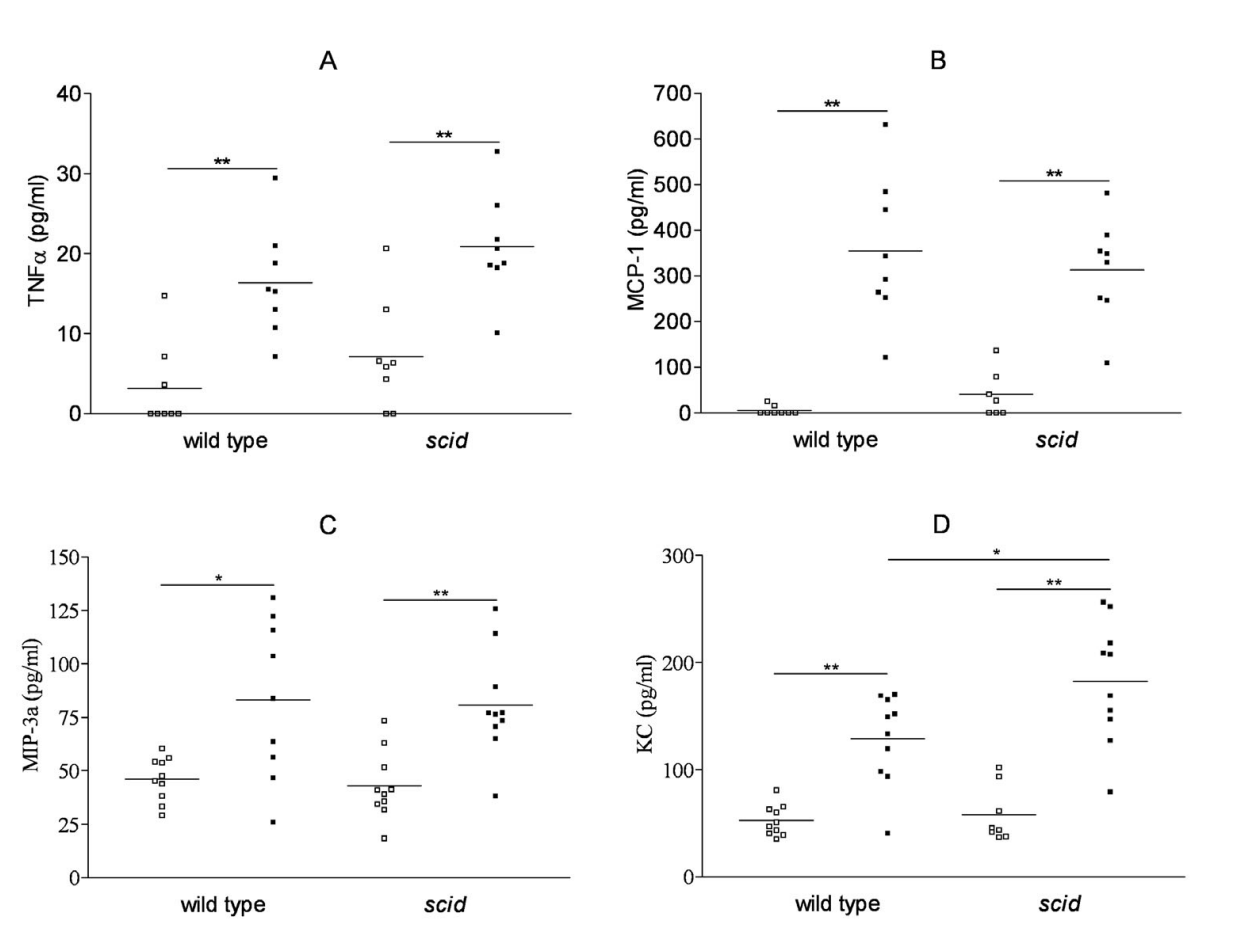
**Protein levels of cytokines and chemokines in bronchoalveolar lavage fluid upon air or cigarette smoke exposure**. Effect of cigarette smoke (CS) exposure for 6 months on the release of cytokines and chemokines in bronchoalveolar lavage fluid of wild type mice and *scid *mice (air-exposed mice: open squares, CS-exposed mice: closed squares). (A) TNF-α, (B) MCP-1, (C) MIP-3α and (D) KC (* p < 0.05, ** p < 0.001). TNF-α (Tumor Necrosis Factor-α) and MCP-1 (Monocyte Chemotactic Protein-1) were measured by Cytometric Bead Array; MIP-3α (Macrophage Inflammatory Protein-3α) and KC (mouse Interleukin-8) were determined by ELISA.

To further decipher the molecular mechanisms involved in the pathogenesis of the CS-induced pulmonary inflammation, several cytokines and chemokines were measured in the BAL fluid by Cytometric Bead Array (TNF-α, MCP-1, IL-10, IL-6, IL-12p70 and IFNg) and ELISA (MIP-3α and KC). At six months, CS-exposure significantly increased the BAL levels of TNF-α, MCP-1, MIP-3α and KC in both *scid *and wild type mice compared to air-exposure (figure 7). Similar levels of TNF-α, MCP-1 and MIP-3α were found in BAL fluid of CS-exposed wild type and *scid *mice, while levels of KC in lavage of *scid *mice were significantly elevated compared to wild type mice upon CS-exposure. The levels of IL-10, IL-6 and IL-12p70 in BAL fluid were low in all groups and did not differ between air- or CS-exposed mice (data not shown). The levels of IFN-γ were below the detection limit in almost all BAL fluid samples, irrespective of mouse genotype or exposure regimen (data not shown).

## Discussion

In this study we show for the first time that chronic cigarette smoke (CS)-exposure for six months (i) increases the number of innate inflammatory cells in BAL and lungs of both wild type and *scid *mice; (ii) increases the number of peribronchial lymphoid follicles in wild type mice in contrast to *scid *mice; and, most importantly, (iii) induces pulmonary emphysema not only in wild type animals, but also in *scid *mice, despite the complete absence of B- and T-lymphocytes in the latter.

A potential role for T-lymphocytes in the development of pulmonary emphysema in human smokers has been suggested, since cytotoxic CD8^+ ^T-cells are increased in the lungs of smokers and have the capacity to cause (perforin- or granzym B-mediated) cytolysis and apoptosis (by Fas-Fas ligand interaction or by caspase activation) of alveolar epithelial cells [6,7,9,10,19-21]. However, the observed correlation between increased T-lymphocytes in the alveolar wall of COPD patients and the extent of emphysema, does not prove a causal relationship. Moreover, considerable variation in the counts of CD8^+ ^T-cells has been described in bronchial biopsies of smokers with chronic bronchitis [22].

Therefore, to examine whether the adaptive immune response, consisting of B- and T-lymphocytes, is actually causing pulmonary emphysema in response to CS, we investigated the effects of subacute and chronic CS-exposure in wild type *Balb/c *mice and in *scid *mice, which lack functional B- and T-cells [13,23]. Chronic CS-exposure induced emphysema in both wild type and *scid *mice. Since *scid *mice have a normal innate immune system, including the epithelial barrier, monocytes/macrophages, neutrophils and highly functional NK cells [24], these experimental findings underline the important role of innate immunity in the development of pulmonary emphysema. After one month CS-exposure, *scid *mice had less neutrophils and macrophages in BAL compared to wild type animals. At six months, however, the number of BAL neutrophils, macrophages and dendritic cells – specialized antigen presenting cells, linking the innate and the adaptive immune system [25] – were even higher in CS-exposed *scid *mice. We speculate that the higher number of neutrophils, macrophages and dendritic cells may indicate a compensatory mechanism for the lack of B- and T-lymphocytes in *scid *mice. Cigarette smoke may activate lung resident cells, including epithelial cells and macrophages, which then release inflammatory mediators, including cytokines (eg TNF-α and KC (mouse IL-8)) and chemokines (eg MIP-3α and MCP-1) [26-29]. We demonstrated increased protein levels of TNF-α, KC, MCP-1 and MIP-3α in BAL-fluid upon CS-exposure, thereby attracting neutrophils, macrophages and dendrititc cells to the lungs. Inflammatory cells might contribute to the development of pulmonary emphysema, since they have the capacity to secrete many elastolytic enzymes, such as neutrophil elastase (by neutrophils) and MMP-12 (by macrophages and dendritic cells). Both proteases have been reported to be important in COPD [4,30,31].

Recently, James Hogg et al. characterized the nature of the small airway obstruction in patients with COPD [11,32]. Interestingly, the progression of COPD was associated with the infiltration of the airway wall by innate and adaptive immune cells that formed lymphoid follicles [11]. Although the cause of the appearance of lymphoid follicles in the later stages of COPD (GOLD stage 3 and 4) is not known, it was speculated that this adaptive immune response develops in response to the microbial colonization and infection known to occur in patients with severe COPD. However, whether these lymphoid follicles are required to develop emphysema in response to CS, is currently not known. Our experimental findings in mice pointed out that (i) chronic CS-exposure indeed induced peribronchial lymphoid follicles in wild type *Balb/c *mice, but that (ii) pulmonary emphysema still developed even in the absence of any lymphoid follicles, as is the case in the *scid *mice. This does not exclude the possibility that the lymphoid follicles could accelerate the decline in lung function in COPD patients or aggravate the extent of CS-induced emphysema in humans. Nevertheless, in this murine model of COPD, the wild type animals did not suffer from more severe emphysema despite the presence of lymphoid follicles.

A possible limitation of the study could be the occurrence of "leakiness" in *scid *mice, which leads to the production of some functional B- and T-cells [33]. However, after chronic exposure to air or CS, only one *scid *mouse out of 13 had serum IgG levels exceeding 20 μg/ml, indicating leakiness. Lastly, the CS-induced emphysema in *scid *mice could theoretically be attributed to the presence of the NK-cell, since an enhanced activity of these innate immune cells has been described in these animals [24]. To test this hypothesis, we determined the expression levels of the 2 major cytolytic effector proteins of NK-cells, namely perforin, a pore-forming protein, and granzyme B, a member of the serine proteinase family [10]. We found elevated RNA levels of granzyme B in *scid *mice compared to wild type animals, but these levels of granzyme B did not augment upon CS-exposure in both strains of mice. Moreover, after chronic CS-exposure, the mRNA levels of perforin did not increase in wild type and *scid *mice, arguing indirectly against the hypothesis that the NK-cell could drive the CS-induced emphysema in *scid *mice (data not shown).

## Conclusion

In this paper, we demonstrated for the first time that chronic exposure to mainstream cigarette smoke can induce pulmonary emphysema in *scid *mice, which lack functional B- and T-cells and which lack peribronchial lymphoid follicles. Since CS-exposure induces a progressive increase in key inflammatory cells of the innate immune system (neutrophils, macrophages and dendritic cells) and augments several epithelium-derived chemokines, these *in vivo *studies suggest that the innate immune system plays a major role in the pathogenesis of COPD and pulmonary emphysema.

## Abbreviations

BAL: bronchoalveolar lavage

COPD: chronic obstructive pulmonary disease

CS: cigarette smoke

DI: destructive index

GOLD: Global initiative for chronic Obstructive Lung Disease

L_m_: mean linear intercept

MCP-1: Monocyte Chemotactic Protein-1 (CCL2)

MIP-3α: Macrophage Inflammatory Protein-3α (CCL20)

MMP-12: matrix metalloproteinase-12

N.S.: not significant

*scid*: severe combined immunodeficiency

TNF-α: Tumor Necrosis Factor-α

## Authors' contributions

AD carried out the design and coordination of the study, performed the quantification of emphysema and lymphoid follicles, carried out the RT-PCR analysis, performed the data and statistical analysis, helped to interpret the data and drafted the manuscript. TM helped to interpret the data and critically revised the manuscript. KB participated in the RT-PCR analysis. ID performed the Cytometric Bead Array analysis and helped to draft the manuscript. KT critically revised the manuscript. GJ critically revised the manuscript. GB participated in the design and coordination of the study, helped to interpret the data and drafted the manuscript. All authors read and approved the final manuscript.
